# The Cybathlon BCI race: Successful longitudinal mutual learning with two tetraplegic users

**DOI:** 10.1371/journal.pbio.2003787

**Published:** 2018-05-10

**Authors:** Serafeim Perdikis, Luca Tonin, Sareh Saeedi, Christoph Schneider, José del R. Millán

**Affiliations:** Defitech Chair in Brain-Machine Interface (CNBI), Center for Neuroprosthetics, School of Engineering, École Polytechnique Fédérale de Lausanne (EPFL), Geneva, Switzerland; UCSF School of Medicine, United States of America

## Abstract

This work aims at corroborating the importance and efficacy of mutual learning in motor imagery (MI) brain–computer interface (BCI) by leveraging the insights obtained through our participation in the BCI race of the Cybathlon event. We hypothesized that, contrary to the popular trend of focusing mostly on the machine learning aspects of MI BCI training, a comprehensive mutual learning methodology that reinstates the three learning pillars (at the machine, subject, and application level) as equally significant could lead to a BCI–user symbiotic system able to succeed in real-world scenarios such as the Cybathlon event. Two severely impaired participants with chronic spinal cord injury (SCI), were trained following our mutual learning approach to control their avatar in a virtual BCI race game. The competition outcomes substantiate the effectiveness of this type of training. Most importantly, the present study is one among very few to provide multifaceted evidence on the efficacy of subject learning during BCI training. Learning correlates could be derived at all levels of the interface—application, BCI output, and electroencephalography (EEG) neuroimaging—with two end-users, sufficiently longitudinal evaluation, and, importantly, under real-world and even adverse conditions.

## Author summary

Noninvasive brain–computer interface (BCI) based on imagined movements can restore functions lost to disability by enabling spontaneous, direct brain control of external devices without risks associated with surgical implantation of neural interfaces. We hypothesized that, contrary to the popular trend of focusing on the machine learning aspects of BCI training, a comprehensive mutual learning methodology could strongly promote users’ acquisition of BCI skills and lead to a system able to succeed in real-world scenarios such as the Cybathlon event, the first international competition for disabled pilots in control of assistive technology. Two severely impaired participants with chronic spinal cord injury (SCI) were trained following our mutual learning approach to control their avatar in a virtual BCI race game. The evolution of the training process, including competition outcomes (gold medal, tournament record), substantiates the effectiveness of this type of training. Most importantly, the present study provides multifaceted evidence on the efficacy of subject learning during BCI training. Learning correlates could be derived at all levels of the interface—application, BCI output, and electroencephalography—with two end-users, longitudinal evaluation, and, importantly, under real-world and even adverse conditions.

## Introduction

Since the first demonstration of the profound clinical potential of brain–computer interfaces (BCIs) [1], the vast majority of studies have pertained to methodological and technical challenges involving experimentation with able-bodied individuals. While these works can be largely credited with the field’s nowadays widely acknowledged versatility and technological maturity, they carry limited evidence regarding its translational impact. Restricting the scope to the case of BCI for communication and control, the number of published works involving end-users in the last 20 years remains to date a modest double-digit figure [2]. As a result, the general concerns about the non-universal usability, robustness, and, especially, the role of training raised by able-bodied user studies [3–7] are even more pressing with regard to end-user populations.

In this study, we investigated the hypothesis that mutual learning is a critical factor for the success of motor imagery (MI) BCI in translational applications. Contrary to a popular trend of focusing almost exclusively on the machine learning aspects of MI training, our hypothesis propounds that a holistic mutual learning training approach grounded symmetrically on all three learning pillars (at the machine, subject, and application level) would be the optimal training apparatus for preparing two end-user participants for the Cybathlon BCI race, the first international BCI competition [8].

Historically, the BCI field has evolved from systems employing simple decoders and relying on the users’ ability to learn to modulate their brain activity (conventionally requiring long training periods) [1,9,10] towards systems deploying elaborate signal processing and pattern recognition algorithms to minimize the user’s training time and to increase information transfer rates [11]. The early approaches exploited classical neurofeedback theories (a form of operant conditioning), tailoring the interface to the needs of assistive scenarios. However, following the artificial intelligence (AI) revolution, it is the latter trend that has greatly dominated the field in the last 15 years. This is substantiated by the fact that more than half of published BCI works research signal-processing and machine-learning methods [12]. Beyond riding the wave of the multidisciplinary progress in AI and data analysis, treating BCI as a primarily neural decoding problem has its roots in two reasons. On the one hand, the emergence of interfaces based on evoked responses (P300, steady-state visually evoked potentials [SSVEP]) as the most efficient BCI solution for communication [13–16] has promoted the use of machine learning because the margin for humans to learn to regulate evoked potentials is considered to be narrow. On the other hand, the machine learning trend has also prevailed in sensorimotor rhythm (SMR)-based BCIs and invasive BCIs that decode different movement parameters. This is grounded in the possibility to tap directly on natural sensorimotor circuits [17]—i.e., to exploit the preexisting correlates of imagined and real movements. However, although machine learning has been critical for major achievements in BCI, “zero-training” and universal BCI remains elusive.

On the contrary, co-adaptive (a term we use interchangeably to mutual learning) interfaces, in which the capacities of both learning agents—the brain and the machine—are accommodated and coordinated, has been very early proposed as a remedy [18] and more recently increasingly adopted and modeled as a training strategy [19–21]. Under this view, successful BCI requires that the user and the embedded decoder engage in a mutual learning process, in which users must learn to generate distinct brain patterns for different mental tasks, while machine learning techniques ought to discover, interpret, and allow a model’s adaptation to the potentially changing individual brain patterns associated to these tasks [22].

Co-adaptation has been studied in depth in the context of invasive and semi-invasive brain–machine interfaces with human and nonhuman primates [19,23,24]. Although it has also been researched in noninvasive SMR-based BCI [21], this body of literature is still characterized by a strong focus on the machine learning side and, in particular, the challenges related to online decoder parameter estimation [25–29]. Evidence that co-adaptive MI BCIs might also be able to promote and increase the ability of the users to voluntary modulate their brain signals (subject learning) is, in fact, scarce, most often indirect and rather inconclusive. Indeed, mutual learning has been claimed mostly on the grounds of adequate and improved BCI classification accuracy [25,27,30–35] or application performances [36,37]. However, those are indirect measures of improved brain signal modulation. Direct evidence of learned SMR modulation at the BCI feature level is, in fact, rare or incomplete, derived in able-bodied populations and not longitudinal [10,23,26,28,38–41]. Notwithstanding a few exceptions of longitudinal and translational studies in which thorough neuroimaging evidence is also provided [9,42], the extent and impact of subject learning effects in noninvasive MI BCI training remain rather disputable.

The third level that we believe promotes acquisition of BCI skills is at the application side, an aspect that is not usually studied in BCI. As for any human–computer interface, we conjecture that the design of the interaction can have a strong impact on how suitable the system is for its user and on how the latter learns to purposefully modulate his/her brain rhythms. To our best knowledge, this is the first time that the influence of the application design on subject learning is quantified in BCI.

**Fig 1:**
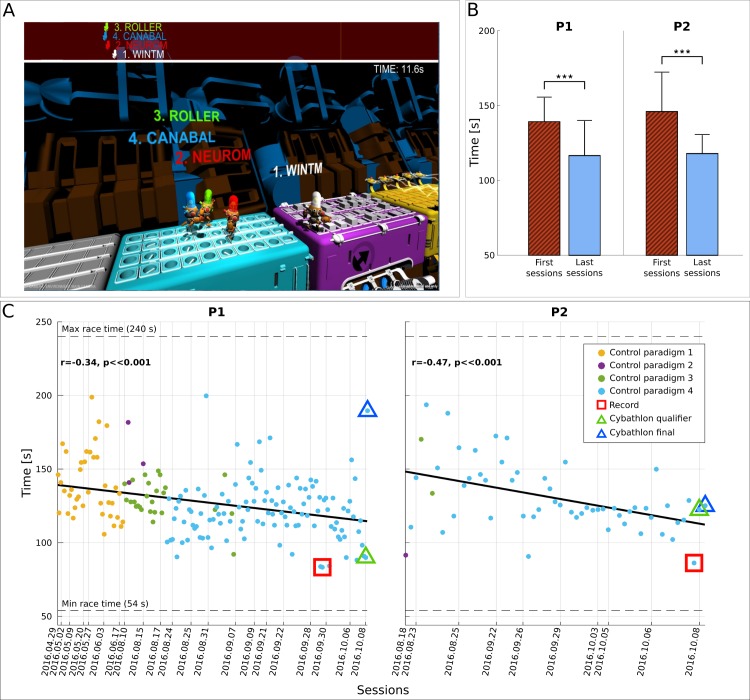
Cybathlon BCI race track and race completion time. **(A)** Standard race track of Cybathlon’s Brain Runners game graphical user interface (BrainRunners, developed for the BCI Race of the Cybathlon 2016 in cooperation of ETH Zurich and Zurich University of the Arts (ZHdK), Switzerland). Pilots need to deliver the proper command in each color pad (cyan, magenta, yellow) in order to accelerate their own avatar. **(B)** Average and standard deviation of race completion time (s) for pilots P1 and P2 in the first (red) and last (blue) four training sessions, including the competition day. Statistically significant differences are shown with two-sided Wilcoxon ranksum tests, (***): *p* < .001. **(C)** Race completion times (s) achieved by pilots P1 and P2 throughout training. The corresponding linear fits and Pearson correlation coefficients (significance extracted with Student *t* test distribution) demonstrate training effects. Dashed horizontal lines illustrate the minimum and maximum race completion bounds of Cybathlon’s BCI race-standard track (perfect control and continuously flawed commands, respectively). Vertical lines indicate the date of each racing session. Marker colors show the control paradigm employed (see Materials and methods). Record performances are highlighted with red squares. The competition performances are highlighted with triangles, green for the qualifier and blue for the final. Fig 1 data is located at https://doi.org/10.5281/zenodo.1205681, https://doi.org/10.5281/zenodo.1205687. BCI, brain–computer interface; ZHdK, Zurich University of the Arts.

According to our hypothesis, endowing our two end-user participants with mutual learning would facilitate the emergence of SMR modulations—supported and complemented (but not overshadowed) by both the use of machine-learning techniques and the refinement of the interaction with the application—that participants can largely sustain even in adverse conditions like the public Cybathlon BCI race. Cybathlon has been the first international para-Olympics for disabled individuals in control of bionic assistive technology (AT) [43], featuring 12 end-users in the BCI race with a level of impairment in the American Spinal Injury Association (ASIA) scale of at least C. Two male individuals (P1 and P2), tetraplegic (ASIA A) and wheelchair-bound as a result of accident-inflicted spinal cord injury (SCI) have been trained to operate our MI BCI for the Cybathlon BCI race as “pilots” of our “Brain Tweakers” team. Coherently to our hypothesis, training followed a mutual learning approach. The BCI race consisted of four brain-controlled avatars competing in a virtual race game called “Brain Runners,” where up to three mental commands (or intentional idling) should be issued on corresponding color-coded track segments (“pads”) to accelerate one's avatar (Fig 1A and S1 Movie). In the absence of BCI input, avatars would walk at medium pace towards the finish line. Timely, correct commands would speed them up and erroneous ones slow them down [8,44].

Our results showcase strong and continuous learning effects at all targeted levels—machine, subject, and application—with both end-users over a longitudinal study lasting several months. This study provides direct evidence on the existence, extent, and impact of subject learning in translational, noninvasive MI BCI. Importantly, these learning effects were achieved under uncontrolled circumstances at the pilot’s homes with minimal expert personnel intervention, while the learned outcome was replicated at a demanding international competition—the first of its kind—under adverse circumstances, where our pilots were able to excel. Although the competition demands have imposed the nature of this study as observational and uncontrolled, we believe our work still pinpoints key ingredients of a successful mutual-learning scheme and contributes to the consolidation of the notion that BCI is a “skill to be learned” [45,46] in the field of electroencephalography (EEG)- and SMR-based interfaces, in which we believe it has been largely neglected.

## Results

### Cybathlon BCI race outcomes

The BCI race discipline of the Cybathlon has provided an ideal opportunity and a unique testbed for the present study on mutual learning. Eleven international BCI teams participated at the event. Each pilot had to mentally control his own avatar in a virtual race game by forwarding three different commands (Fig 1A). The race completion time was the criterion for winning the game. The competition consisted of two phases: Qualifiers and Finals. The four pilots who marked the best completion times in the ensemble of Qualifiers advanced to Final A, the second-best group of four pilots proceeded to Final B and the remaining competitors were eliminated for the rest of the tournament. The first three pilots in Final A received the gold, silver, and bronze medals, respectively. The official results of the Cybathlon BCI discipline are reported in Table 1. In order to appreciate the race completion time of the BCI pilots, perfect control would make the avatar finish in 54 s, continuous wrong commands would result in 240 s, and a system not delivering any command would yield 162 s.

**Table 1:** Cybathlon BCI race results. The table presents the race completion times of all competing pilots in the Qualifiers and in Final A and B races of the Cybathlon BCI race, and the pilots’ final rankings.

Team (pilot)	Completion Time (s)	Rank
**Qualifier**		
Brain Tweakers (P1)	90	1
Brain Tweakers (P2)	123	2
BrainGain	135	3
BrainStormers	146	4
Athena-Minerva	148	5
OpenBMI	149	6
Neurobotics	161	7
NeuroCONCISE	165	8
Mahidol BCI	167	9
Ebrainers	186	10
MIRAGE91	196	11
ENS Lyon	N/A	Raced out of competition—Pilot ineligible
**Final A**		
Brain Tweakers (P2)	125	1
BrainGain	156	2
BrainStormers	161	3
Brain Tweakers (P1)	190	4
**Final B**		
Neurobotics	132	5
NeuroCONCISE	136	6
Athena-Minerva	146	7
OpenBMI	149	8

**Abbreviation:** BCI, brain–computer interface.

P1 qualified with 90.1 s, a performance that set the competition record, almost 32 s ahead of the second-best time belonging to our second pilot, P2 (122.5 s). In the final, the third-best competition time (125.3 s) was made by P2 to win the gold medal. The closest times belonging to the pilots of other competing teams throughout the tournament were 132, 135, 136, and 146 s. P1 experienced a momentary loss of BCI control and had to compromise with the fourth place in the final (189.8 s).

### Primary outcome

The Cybathlon racing application naturally determined the race completion time as the primary outcome of our study. Fig 1B shows that our training procedure reduced the race completion time of P1 from 139.2 ± 16.1 s (*N* = 18, first four racing sessions) to 116.5 ± 23.2 s (*N* = 34, last four racing sessions, including the competition day) and similarly for P2 from 145.9 ± 26.1 s (*N* = 22) to 117.9 ± 12.5 s (*N* = 21). Both these improvements are statistically significant (*p* < .001, two-sided Wilcoxon ranksum tests). The race completion times of our pilots throughout training (Fig 1C) averaged 126.9 ± 21.3 (*N* = 182) s for P1 and 130.3 ± 22.9 (*N* = 57) s for P2, with all-time records of 83.3 and 86.3 s, respectively. Significant negative Pearson correlations between race time and (chronological) race index establish the existence of a significant training effect on race time (Fig 1C, P1: r = −0.34, *p* < .001, *N* = 182; P2: r = −0.47, *p* < .001, *N* = 57). P1 achieved slightly better average and record performances, while P2 exhibited superior stability, having race time standard deviation of 12.9 s in the last 5 sessions (*N* = 28), as opposed to 20.6 s for P1 (*N* = 50).

### BCI performances

**Fig 2:**
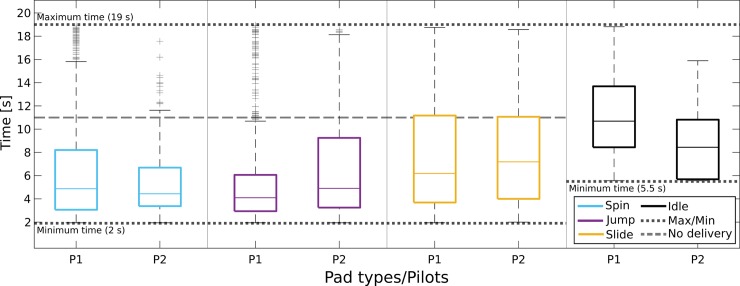
Pad crossing time throughout training. Boxplots of pad crossing time (s, time spent on each pad) for pilots P1 and P2, for all types of pads (cyan for spin, magenta for jump, yellow for slide, black for idling) and for all races (training and competition). The box edges signify the 75th (top) and 25th (bottom) percentiles and the colored horizontal line the median of the corresponding distribution. The whiskers extend to the largest and smallest nonoutlier values. Outliers are marked with black crosses. Dotted horizontal lines illustrate the minimum (accurate and precise BCI input), maximum (continuously erroneous BCI input), and no-delivery (unresponsive BCI, avatar goes at “base” speed) crossing times for the different pad types. The dashed line corresponds to the no-delivery time in the spin, jump, and yellow pads. Fig 2 data is located at https://doi.org/10.5281/zenodo.1205693. BCI, brain–computer interface.

We employ “pad crossing time” as the optimal index to evaluate BCI performance, since it assesses BCI command delivery accuracy and speed in a single metric, while also better reflecting the task at hand [47]. The more widely used metric of BCI command accuracy is also provided below. Fig 2 illustrates that the high-yielding application performances come as a result of our pilots' ability to adequately master all four individual subtasks required by the application: the intentional control (IC) ability to deliver the correct command on the action pads (spin, jump, slide) and the intentional non-control (INC) ability to “rest/idle” on the white pads [48–50]. The illustrated median pad crossing time performances (for P1/P2) across all races (training and competition) were 4.9/4.4 s (*N* = 853/205) for spin, 4.1/4.9 s (*N* = 766/198) for jump, and 6.2/7.2 s (*N* = 463/196) for slide, which compare favorably to the lower bound (2 s) while lying far away from this metric's imposed upper bounds (11 s if no mental command is forwarded, 19 s for continuously erroneous command delivery) for all active command types. Remarkably, a similar argument can be made for the INC ability. The median crossing time of white pads was 10.7 s and 8.4 s for P1 (*N* = 510) and P2 (*N* = 151), respectively—far below the worst-case scenario of 19 s and closer to the optimum of 5.5 s. It is also worth noting that the average pad crossing time correlates with the primary outcome of race completion time (P1: r = 0.79, *p* < .001, *N* = 162; P2: r = 0.92, *p* < .001, *N* = 45), showing that improvements in BCI performances have driven the application performance enhancement. Furthermore, average pad crossing time improves with training, as shown by its correlation with the run index (P1: r = −0.40, *p* < .001, *N* = 162; P2: r = −0.43, *p* = .003, *N* = 45).

**Fig 3:**
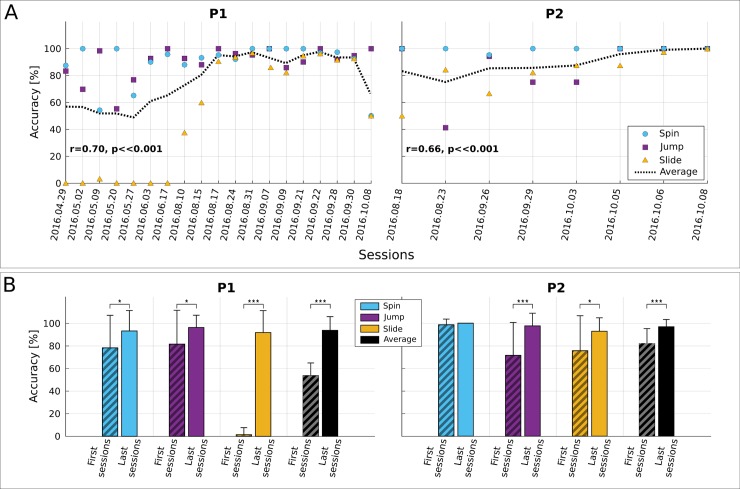
BCI command accuracy. For the sake of clarity, accuracy in the figure is reported per session instead of per race. **(A)** Average within-session BCI command accuracy (in percent) for pilots P1 and P2. Spin command accuracy shown in cyan, jump in magenta, and slide in yellow. The dashed black line shows the overall accuracy (average of individual command accuracies) in a session. The Pearson correlation between the overall command accuracy and the chronological race index is also reported (significance tested with Student *t* test distribution). **(B)** Average and standard deviation of BCI command accuracy (in percent) for pilots P1 and P2 for all command types (cyan for spin, magenta for jump, yellow for slide) and overall (black) in the first and last four training sessions, including the competition day. Statistically significant differences are shown with two-sided Wilcoxon ranksum tests, (*): *p* < .05, (***): *p* < .001. Fig 3 data is located at https://doi.org/10.5281/zenodo.1205695, https://doi.org/10.5281/zenodo.1205699. BCI, brain–computer interface.

Fig 3A verifies increasing trends of command accuracy for both pilots and all command types. This can be quantified by significant positive correlations of the overall accuracy to the (chronological) race index (P1: r = 0.70, *p* < .001, *N* = 162 races; P2: r = 0.66, *p* < .001, *N* = 45 races). Fig 3B showcases that the average total accuracy of P1 improved significantly from 53.8% (*N* = 18) to 93.8% (*N* = 41) and that of P2 from 81.9% (*N* = 24) to 96.8% (*N* = 21) (P1 and P2: *p* < .001 with two-sided Wilcoxon ranksum tests). Both pilots exhibited significant command accuracy increase in all individual tasks (the only exception being the spin command for P2, with stable accuracy). In the same sessions, the percentage of pads crossed without a false positive increased from 19.2% to 29.1% for P1 and slightly deteriorated for P2 (from 34.3% to 31.0%). Like the pad crossing time, command accuracy correlates with the race completion time (P1: r = −0.62, *p* < .001, *N* = 162 races; P2: r = −0.57, *p* < .001, *N* = 45 races).

### Neurophysiological evidence of subject learning

Our training approach targeted sessions twice a week and initially involved “offline,” open-loop BCI training, in which our pilots performed a number of MI tasks without observing real-time feedback so as to identify the optimal tasks and calibrate the BCI. This was followed by “online,” closed-loop BCI feedback training allowing the users to gradually optimize the modulation of their brain rhythms [51]. Finally, race training allowed our end-users to familiarize with the actual BCI application demands while further improving their BCI skills. BCI recalibration was performed only twice per pilot (P1: 30/06/2016 and 14/09/2016; P2: 11/08/2016 and 08/09/2016). Table 2 presents the selected spatiospectral features (bands and Laplacian channels).

**Table 2:** Features selected for mutual learning. The table presents all the spatio-spectral features selected for the BCI classifiers trained throughout our pilots’ mutual learning process. Each feature refers to a specific frequency band (2 Hz resolution) and EEG channel location according to the international 10–20 system.

P1			P2		
Date	Feature		Date	Feature	
	Location	Band (Hz)		Location	Band (Hz)
30/06/2016	C1	22	11/08/2011	C1	26
	C1	24		C1	28
	Cz	12		C1	30
	Cz	20		Cz	30
	Cz	22		C2	26
	Cz	24		C2	28
	C2	18		C2	30
	C2	20		CPz	26
	CP3	20		CPz	28
	CP3	22	08/09/2016	C1	32
	CP3	24		Cz	28
	CPz	18		Cz	30
	CPz	20		Cz	32
14/09/2016	FC4	28		CP3	30
	FC4	32		CP3	32
	Cz	10		CPz	24
	Cz	12		CPz	26
	Cz	20			
	Cz	22			
	Cz	24			
	C4	26			
	C4	28			
	C4	30			
	CP3	22			
	CP3	24			

**Abbreviation:** EEG, electroencephalography.

**Fig 4:**
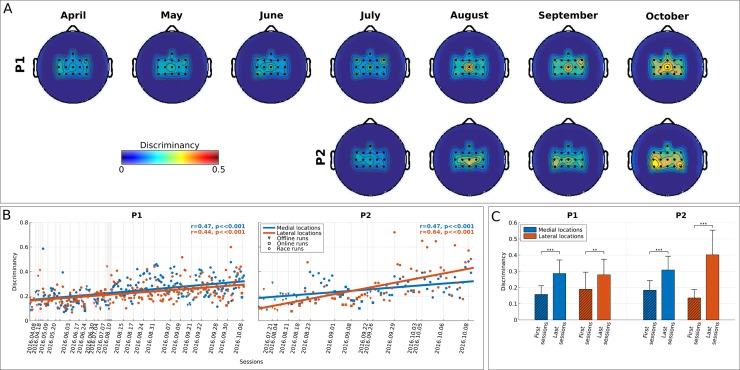
BCI feature discriminancy. **(A)** Topographic maps of discriminancy per training month on the 16 EEG channel locations over the sensorimotor cortex monitored. Bright color indicates high discriminancy between Both Hands and Both Feet MI tasks employed by both pilots (P1 top, P2 bottom). The discriminancy of each channel is quantified as the Fisher score of the EEG signal's power spectral density distributions for these two mental classes in the high β-band (22–32 Hz) within each run. Each map illustrates local Fisher scores (with interchannel interpolation) averaged over all runs within the supertitled month. **(B)** Average medial (blue, channels: FCz, Cz, CPz) and lateral (red, channels: FC3, C3, CP3, FC4, C4, CP4) discriminancy for all performed offline, online, and racing runs of pilots P1 and P2. The corresponding linear fits and Pearson correlation coefficients (significance tested with Student *t* test distribution) are reported to indicate training effects. Vertical dashed lines indicate the training session during which each run took place. **(C)** Average and standard deviations of medial region (blue) and lateral region (red) discriminancy within the first and last four runs of training for pilots P1 and P2. Statistically significant differences are shown with two-sided Wilcoxon ranksum tests, (**): *p* < .01, (***): *p* < .001. Fig 4 data is located at https://doi.org/10.5281/zenodo.1205702, https://doi.org/10.5281/zenodo.1205704, https://doi.org/10.5281/zenodo.1205708. BCI, brain–computer interface; EEG, electroencephalography; MI, motor imagery.

Fig 4A demonstrates that our incremental mutual learning procedure has been very effective in bringing up an emerging SMR pattern (high β-band, 22–32 Hz) for both pilots, coherent with both hands MI (lateral, electrodes FC3, C3, CP3, FC4, C4, CP4 of the 10–20 EEG system) and both feet MI (medial, electrodes FCz, Cz, CPz) locations of the sensorimotor cortex (see also S1 Fig for discriminancy maps in higher-frequency resolution). Fig 4B further substantiates a significant enhancement trend of these patterns' discriminancy over runs (P1, *N* = 214: r = 0.47, *p* < .001 for medial and r = 0.44, *p* < .001 for lateral locations; P2, *N* = 79: r = 0.47, *p* < .001 for medial and r = 0.64, *p* < .001 for lateral locations), accounting for considerable, statistically significant increase for both pilots and locations between the first and last four sessions (Fig 4C).

The overall discriminancy of our pilots’ SMRs (average of medial and lateral locations for P1, lateral for P2) correlates well with the total command accuracy (P1: r = 0.56, *p* < .001, *N* = 162; P2: r = 0.37, *p* = .013, *N* = 45), the average pad crossing time (P1: r = −0.42, *p* < .001, *N* = 162; P2: r = −0.45, *p* = .002, *N* = 45), and the race completion time (P1: r = −0.39, *p* < .001, *N* = 162; P2: r = −0.29, *p* = .0056, *N* = 45). Hence, increased SMR modulation (discriminancy) seems to be crucial for enhanced BCI and, through the latter, also application performances.

**Fig 5:**
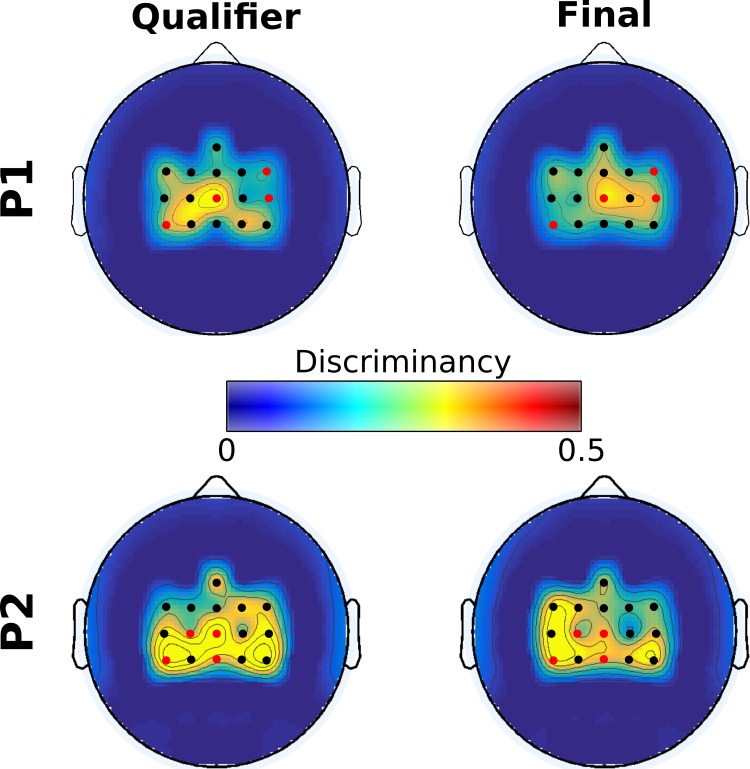
BCI feature discriminancy for pilots P1 and P2 in the Cybathlon. Topographic maps of discriminancy per Cybathlon race on the 16 EEG channel locations over the sensorimotor cortex monitored. Bright color indicates high discriminancy between Both Hands and Both Feet MI tasks employed by both pilots. The discriminancy of each channel is quantified as the Fisher score of the EEG signal's power spectral density distributions for these two mental classes in the high β band (22–32 Hz), on this channel. Each map illustrates local Fisher scores (with interchannel interpolation) in the supertitled race. Selected channels are indicated by red color. Fig 5 data is located at https://doi.org/10.5281/zenodo.1205711. BCI, brain–computer interface; EEG, electroencephalography; MI, motor imagery.

Fig 5 sheds light on the neurophysiological basis of P1’s poor performance in the final. It can be seen that P1’s inability in this particular race to deliver any command associated to the Both Hands MI task (spin, slide) has been accompanied by the disappearance of this task’s identified EEG correlates, namely the β-band SMR discriminancy in locations contralateral to the dominant right hand, selected for the classifier used in the competition (CP3, Table 2). On the contrary, pilot P2 largely maintained the same brain pattern in both competition races, even increasing the strength of medial modulation in the final (channels Cz and CPz, both channels were selected for the classifier used in the competition).

### Effects of the application in BCI control and learning

The BCI’s configuration (choice of appropriate values of some hyperparameters, such as the decision threshold) and the application control paradigm have substantially benefited from our pilot's input, following a user-centered approach in BCI design. In particular, end-user feedback has largely shaped our BCI’s control paradigm (see Materials and methods). As shown in Fig 1C, early attempts with a 3-class BCI (paradigm 1) severely compromised the total command accuracy (Fig 3A), which is reflected in the high race completion times during this period. Supporting only two commands (paradigm 2) was clearly suboptimal, since the application demands could not be fully met with a binary input. Thus, while the two separable MI tasks (kinesthetic both hands and feet MI for both our pilots) were directly mapped to the spin and jump avatar actions, two different solutions were evaluated for the slide command. Paradigm 3 would make the avatar slide after a configurable inactivity period. Paradigm 4 would trigger sliding when two commands of different type were forwarded within a configurable timeout [44].

**Fig 6:**
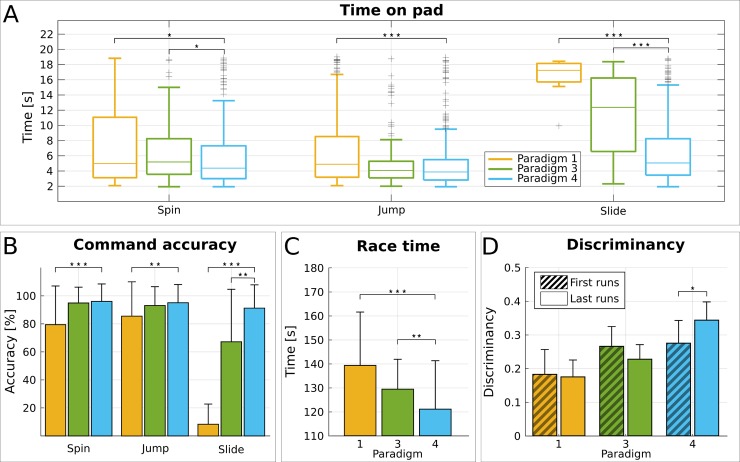
Effects of the control paradigm. **(A)** Boxplots of pad crossing time (s) for pilot P1 and all pad types (spin, jump, slide) and control paradigms 1 (yellow), 3 (green), and 4 (cyan). The box edges signify the 75th (top) and 25th (bottom) percentiles and the colored horizontal line the median of the corresponding distribution. The whiskers extend to the largest and smallest nonoutlier values. Outliers are marked with black crosses. **(B)** Average and standard deviation of BCI command accuracy (in percent) for pilot P1 and all command types (spin, jump, slide) and control paradigms 1 (yellow), 3 (green), and 4 (cyan). **(C)** Average and standard deviation of race completion time (s) for pilot P1 and control paradigms 1 (yellow), 3 (green), and 4 (cyan). **(D)** Average and standard deviation of overall feature discriminancy (medial and lateral locations) in the first and last 10 runs for pilot P1 and control paradigm 1 (yellow), 3 (green), and 4 (cyan). Statistically significant differences are shown with two-sided Wilcoxon ranksum tests (for the sake of clarity, only with respect to paradigm 4). (*): *p* < .05, (**): *p* < .01, (***): *p* < .001. Fig 6 data is located at https://doi.org/10.5281/zenodo.1205810, https://doi.org/10.5281/zenodo.1205818, https://doi.org/10.5281/zenodo.1205822, https://doi.org/10.5281/zenodo.1205828. BCI, brain–computer interface.

The latter protocol has been shown to be significantly superior for P1 (who executed enough races with each control paradigm) in terms of the median time spent on yellow pads (Fig 6A) that reduced significantly (*p* < .001, two-sided Wilcoxon ranksum test) from 12.4 s (*N* = 83) with paradigm 3 to only 5.1 s (*N* = 363) with paradigm 4. Simultaneously, the slide command accuracy increased significantly (Fig 6B, *p* = .0019, two-sided Wilcoxon ranksum test) from 67.2% ± 37.8% (*N* = 26) to 91.2% ± 17.0% (*N* = 94). This naturally led to important reduction of the race completion time with paradigm 4 (Fig 6C, 121.2 ± 20.1 s, *N* = 114 against 129.5 ± 12.4 s, *N* = 26, *p* = .0039, two-sided Wilcoxon ranksum test), which was finally selected for the competition. While, as shown below, this improvement must be confounded with subject learning effects, an immediate effect of the control paradigm on performance can also be established by comparing the last 10 races with paradigm 3 against the 10 first ones with paradigm 4 (130.1 ± 17.2 s to 112.4 ± 15.1 s, *p* = .0312 with two-sided Wilcoxon ranksum test). Importantly, during these races, P1 alternated between paradigms 3 and 4 (Fig 1A), and for this reason, we cannot expect strong subject learning effects.

The subject and machine learning processes have been always thought to affect each other [18,20,52]. Interestingly, we can show that the involvement of the application in the learning process also creates bidirectional interactions. Specifically, Fig 6D shows the feature discriminancy of the first and last 10 runs of the training periods with the three control paradigms. Interestingly, the discriminancy significantly increased only in the case of control paradigm 4 (0.27 ± 0.07 to 0.34 ± 0.05, *p* = .045, two-sided Wilcoxon ranksum test), while no difference (or even a reduction) is reported for the other two paradigms. Results suggest that the refinement of the control paradigm might have had a critical role in facilitating subject learning.

## Discussion

This study investigates the hypothesis that mutual learning is a critical underlying factor for the success of MI BCI in translational applications. We deemed the Cybathlon to be a unique opportunity to probe this hypothesis, considering the unprecedented participation of 12 end-users in a competitive scenario and the possibility of longitudinal usage of a real BCI application, as well as the harsh training at the users’ homes and the adverse operation conditions imposed by the competition.

This manuscript employs the term “mutual learning” in a wide sense. We consider all training paradigms involving closed-loop BCI control and BCI algorithms in need of parameter estimation to be candidate mutual learning schemes. Effectively, we only exclude open-loop and pure neurofeedback approaches (where there is no BCI decoder). On the contrary, we take into account both “conventional” MI training protocols [51], which alternate the machine- and subject-learning procedures, and “co-adaptive” protocols, in which online feedback training takes place simultaneously with online decoder adaptation [21].

The main contribution of this work is the provision of quantitative evidence regarding the possible extent of operant subject learning in longitudinal MI training, how it can drive both BCI and task performance, and how it can be facilitated by the refinement of the application control paradigm. The significance of our results is that by showcasing the importance of supporting all three mutual learning pillars—subject, machine, and application—we encourage a much-needed shift of focus of the employed training paradigms towards the parallel promotion of operant conditioning effects and the consideration of application designs, to complement the progress in machine learning [12] and fuel the design of next-generation translational BCI training [47,53].

In order to mitigate to some extent the absence of control in our study, a short questionnaire concerning information on the user training aspects of our competitors (see Materials and methods) was addressed to all 10 competing teams and replied to by 6 of them (S1 Table). In support of our hypothesis, it seems that none of these teams invested considerably in the facilitation of subject learning and were mainly concentrated on the machine learning side.

In fact, a recent study analyzing the results of the Cybathlon BCI race states that it was not possible to identify any factor (hardware, signal processing, machine learning, and pilot’s conditions) explaining the performance [8]. So we believe that it is our truly mutual learning protocol that accounts for the results of the competition.

### Subject learning in MI BCIs

It is critical to comment on the reasons why we perceive the indications provided so far in the BCI literature regarding subject learning to be insufficient. To begin with, subject learning in online MI BCI is most often hypothesized to occur “by default” in analogy to neurofeedback training [1,46,54,55]. However, this extrapolation is by no means straightforward, as neurofeedback typically exerts lesser demands, requiring control over predefined brain signals by direct observation [56], while SMR BCIs are complex pattern-recognition systems feeding back transformations of multivariate brain activity [46]. In fact, as mentioned previously and further developed below, evidence of subject learning in BCI is scarce. It is important to note that we wish not to challenge the theory that BCI and neurofeedback learning share the same underlying plasticity mechanisms [46] but, on the contrary, substantiate it by providing solid experimental evidence.

Another similar, overstated extrapolation regards evidence from invasive and semi-invasive BCI, where learning and co-adaptation have been well documented [19,23,24,57–59]. Again, given the significant differences in terms of signal-to-noise ratio (SNR) and other basic characteristics of (semi-)invasive and noninvasive signals, these studies cannot be said to certainly generalize to noninvasive MI BCI.

Interestingly, users have reported reaching a state of proficiency through learning where BCI control becomes “automatic,” as they no longer need to engage explicitly in MI [23,40–42,50,60,61]. This was also reported by our pilot P2 [44]. However, such claims are rather qualitative and do not constitute hard evidence for the existence of subject learning. We argue that this effect must still be accompanied by increasing and consolidated separability of the brain patterns in order to drive BCI performance upwards.

It is mainly the lack of quantitative evidence of subject learning in EEG SMR BCI that is problematic. Firstly, works where users acquired BCI control (able-bodied [3,25] and end-users [40,41,51,62,63]) do not report any learning metric over time. Secondly, other training studies claim learnability in BCIs only on the grounds of improved online classification accuracy [9,27,30,31,33,35,51] or application performances [36,37]. However, accuracy and application-specific performance metrics do not imply improvements of brain signal modulation. Better performance could be due to decoder recalibration [29], re-parameterizations of the BCI, and the application and adoption of better mental strategies [9,64,65], among other factors. Hence, we consider that deriving some index of neuroimaging-based separability at the feature level in order to quantify the user’s BCI aptitude (and its evolution over time) is a sine qua non prerequisite for corroborating the existence of subject learning [46].

Evolution of SMR modulation has been reported, but these studies suffer from certain shortcomings. Some works find no evident learning effects at the neural correlate level [25,34]. Other studies have reported emergence of such SMR modulations [26,28,38,39], but given the short number of experimental sessions they carried out, the observed neurophysiological patterns might only be indicative of transitory effects rather than consolidated subject learning. Our previous work has even reported a short-term decrease in feature discriminancy during adaptive spelling [29].

The most complete evidence of subject learning with obvious translational implications is offered in [9], [10], and [42]. These works report on longitudinal training and involve end-users. Furthermore, [10] and [42] substantiate learning effects with event-related desynchronization/synchronization (ERD/ERS) maps and SMR topographies, respectively, over 3–4 time points throughout the training period. Nevertheless, these works do not explicitly relate induced brain rhythm changes to BCI performance or show that SMR improvements were consistent and continuous.

### Evidence of mutual learning during training for the Cybathlon

The present manuscript provides results that address such limitations in the literature on mutual learning with respect to its subject learning component while also offering novel insights on a possible role of the application on subject learning.

From the machine learning perspective, our results clearly show a positive correlation of the BCI performances (BCI decoding accuracy and pad crossing time) to chronological runs for both users (Fig 3A). This positively influences the application outcomes with a decrease of the race time over the whole training period, as BCI performances correlate significantly with race time improvement (Fig 1C). As already mentioned, BCI was recalibrated only twice for each user (Table 2), but possible new classifiers were trained after every session with the new recorded data and the simulated performances were evaluated. In such an iterative process, most of the classifiers were discarded during the training period due to similar performances. One might argue that such an infrequent BCI recalibration contradicts the mutual learning hypothesis. However, this approach is substantiated by the fact that BCI decoding achieved high-level accuracy (Fig 3A) for both users after the initial recalibrations. Thus, we had assumed that the machine learning model was sufficiently optimized.

We have selected feature discriminability as the index to assess the effects of subject learning at the neurophysiological level because it directly measures users’ ability to modulate different SMRs. In this respect, subject learning is substantiated by the gradual increase of feature discriminability (Fig 4). The reported correlations between discriminancy, BCI performances, and race time establish the impact of subject learning within the mutual learning scheme.

Several indications assert that the learning effects observed here correspond to instrumental learning, as traditionally hypothesized [46]. First, SMR discriminancy increase is shown to be gradual and smooth for both users (Fig 4), as expected for neurofeedback operant conditioning. No apparent “breakthroughs” are evident, which could support the only likely alternative hypothesis, that of the employment of better mental strategies sparking immediate, rather than gradual, improvements [9,64].

At the level of mechanisms, our feedback training design has respected the neuropsychological basis of operant conditioning, namely immediacy and contingency of the visual feedback to the targeted brain rhythms. Indeed, during races, BCI commands always coincide with the presence of SMR, which has to be sufficiently large for the BCI to reach the decision threshold. Thus, although the BCI did not deliver a command to the avatar every time the pilot generated an SMR, the opposite holds: whenever the BCI delivered it, the pilot was eliciting an SMR. Another clear manifestation of the instrumental nature of subject learning is the fact that, as shown in Table 2, the brain features that responded to training were among those selected for classification and feedback provision.

According to our hypothesis, the third pillar of mutual learning, the application design, can play a critical role. In this regard, our results show that the subject learning has substantially benefited from the refinement of the control paradigm according to P1’s suggestions. This new control paradigm seems to have directly influenced his ability to learn how to modulate his brain patterns (Fig 6D). In fact, the user not only exhibited a general improvement of the features’ separability from the initial design to the final one (from control paradigm 1 to 4, Fig 6D) but also a significant positive trend only in the case of the last control paradigm. In the other cases, discriminancy remains stable (or even decreases) over time.

It is interesting to note that, while one might have expected a stabilization of feature discriminancy once BCI command accuracy saturated to high levels (Fig 3A), it continues to increase for both pilots even after the last recalibration (Fig 4B). This might be explained by the fact that the Cybathlon application imposed high demands not only on command accuracy but also on delivery speed, which had further margins of improvement (Fig 2A). Our results are in line with the emerging belief about the need for more stimulating BCI training contexts [47,53,66].

### Limitations

The present study suffers certain limitations, the main one being that it was conceived as an uncontrolled, observational study. Nevertheless, we can rely on our competitors as a fair control group because they have essentially adopted a training methodology mainly based on machine learning, as per the results of the questionnaire (S1 Table), while we followed a more holistic mutual learning methodology. Indeed, their approach involved frequent classifier recalibration and feature re-selection, as well as training protocols that were relatively short and/or not particularly intense. Of note, the differences in machine-learning methods of all participating teams were too subtle to explain the competition outcomes according to the organizers [8].

A second important limitation regards the fact that we report on only two individuals. Still, the fact that both participants exhibited the same training effects and comparable performances makes us confident that our conclusions should generalize, at least to populations with similar clinical profiles.

Due to the logistical constraints of the Cybathlon, the available neuroimaging data was limited to 16 EEG channels. Thus, we have not been able to investigate more deeply the brain plasticity effects induced by subject learning. However, it must be noted that the extracted SMR discriminancy index would be the primary descriptor of learning anyway, since the latter can only be an instance of neurofeedback operant conditioning if learned brain activity modulation happens with respect to the same neural activity that is fed back to the user (in our case, SMRs on selected channels and bands).

Unsatisfactory robustness of our BCI, especially for P1, is another important shortcoming. Lack of robustness is a well-known issue of all BCI paradigms and has been associated to the nonstationarity of brain signals [18,25,27]. As shown, although P1 showcased better average performance, he also exhibited higher variability than P2. This effect, also reflected in our pilot's competition outcomes in which P1 set the record time but was unable to replicate it a few hours later, suggests that stability (robustness) is at least as crucial as performance (effectiveness) for optimal BCI control. We have shown that loss of control for P1 in the final was the result of the disappearance of the SMR modulations normally induced by his Both Hands MI (Fig 5). Various psychological factors (such as motivation, attention, and stress) have been implicated in these negative effects [2,47,53,66–68], which unfortunately are quite frequent in MI BCI operation. On the other side, P2 seemed to have gained stability along his training. We speculate that, although not the only factor, longitudinal mutual learning could help increase robustness.

### Mutual learning: Lessons and recommendations

We believe that the present study pinpoints critical elements of a successful mutual learning methodology, in spite of the aforementioned limitations and although such recommendations are to some extent speculative. We denote that our training apparatus, which certainly falls under the category of “conventional” MI BCI training protocols relying on visual feedback training on top of initial machine calibration with spontaneous SMRs, has been very similar to the one we have applied in our previous work [51]. There, a considerable number of end-users failed to acquire BCI control, especially those without distinct spontaneous SMRs at training onset. We postulate it is mostly the small, but potentially crucial, differences between that and the present study that might explain the different outcomes.

First and foremost, our previous study imposed up to 10 training sessions with low intensity (maximum twice but mostly once per week or even every other week) before a performance criterion could be reached and allow a user to proceed with application control. Our Cybathlon data, especially those of P1 (Fig 4A), show that this amount of training would be insufficient to develop their full BCI potential, even despite increased training intensity. The experiences shared by our Cybathlon competitors point towards the same direction.

Second, training with the BCI application rather than towards it—like in [51] and most other studies—had a profound impact, as shown in Fig 6, for both application performances and subject learning. This might be related to a need for getting accustomed to the actual application demands [47] but probably also to increased user motivation [68] provided both by the gaming application and the goal of participating in an international competition [8]. Based on this experience, we believe that novel motivational paradigms should consider incorporating the element of “competition” (for instance, training with multiplayer games). Related to this, another contributing factor to successful SMR enhancement might have been that we have implemented an “incremental learning” approach as advocated in [53] and shown in [69], where open-loop, closed-loop, and application training (tasks of increasing difficulty) followed each other throughout training. S2 Fig illustrates how the SMR brain patterns of both participants for these different stages resemble each other but are enhanced in magnitude of discriminancy, suggesting that they both gradually adapted to the increasing task demands.

Last but not least, we postulate that, despite current opinion considering this potentially detrimental to BCI accuracy, infrequent recalibration of the BCI has also been beneficial to the subject learning side (Fig 4) while still adequately accommodating the machine learning side of our mutual learning scheme (Fig 2 and Fig 3). Frequent or continuous recalibration, especially in case it is accompanied by re-selecting the classifier’s features, creates a situation in which the subject’s learning could be hindered by the demand to adapt to a continuously changing decoder [29,70]. Since the plasticity/stability dilemma with respect to MI BCI co-adaptation has not been adequately studied so far [21,52], we believe that a parsimonious approach eventually trading off decoding accuracy in the short term in order to better fulfill the subject learning objective in the long term, as done here, is preferable. Comparison with our competitors’ known strategies are in agreement with this assessment. Such fine-tuning of the machine- and subject-learning demands warrant further research and might unlock the full potential of MI BCI co-adaptation.

In conclusion, the Cybathlon 2016 provided the ideal framework to implement and evaluate the effects of longitudinal mutual learning, which allowed us to showcase continuous and consolidated learning not only on the machine side (which is regularly well documented) but also—and most importantly—on the user side, as well as an effect of application training on subject learning. Furthermore, the Cybathlon motivated the recruitment of two end-user participants and the involvement of a real BCI application operated in real-world circumstances, which advocates translational implications of our findings. Importantly, all learning indices (the subject’s and the machine’s), as well as application performances, can be shown to correlate with the amount of training and with one another, which establishes that the individual subject and machine learning improvements are not irrelevant but actually drive the enhancement of BCI-actuated application control.

## Materials and methods

### Ethics statement

This study has been approved by the Cantonal Committee of Vaud (VD, Switzerland) for ethics in human research (CER-VD) under protocol number PB_2017–00295 (20/15 CCVEM).

### Study design

The objective of this study was to train two end-users with severe motor impairments following a mutual learning approach so as to control the Brain Runners BCI application and participate in the Cybathlon BCI race. In pursuit of this goal, the Brain Tweakers have applied the ensemble of BCI machine-learning and signal-processing methods, control paradigms, and mutual learning protocols developed in our lab. The competition and logistical constraints have imposed the nature of this study as an uncontrolled (observational) and longitudinal two-case study.

Our inclusion criteria necessarily coincided with those of the Cybathlon BCI race: minimum age of 18, sufficient cognitive and communication abilities to understand the discipline’s rules, and tetraplegia or tetraparesia as a result of SCI, amyotrophic lateral sclerosis (ALS), or another lesion, quantified with a score of “C” or above on the ASIA impairment scale. The exclusion criteria consisted of cardiac pacemakers, cyber-sickness, and epilepsy. All EEG and race time data collected have been included into our statistical analysis, and no outliers have been defined.

The race completion time is naturally the study’s primary outcome. Each individual training run or race is an evaluation end point. We additionally define a number of essential secondary outcomes evaluating our mutual learning protocol’s machine learning effects (i.e., the time spent on each pad type and the BCI command delivery accuracy) and subject learning effects (SMR brain pattern discriminancy).

### Pilots

Both our pilots—48-year-old P1, injured in December 1989, and 30-year-old P2, injured in May 2003—have sustained complete lesions at level C5–C6 and have scored “A” (Complete injury—No motor or sensory function is preserved in the sacral segments S4 or S5) in the ASIA impairment scale. Both end-users were under medication for the treatment of spasms and other symptoms related to their medical condition. Residual motor abilities included, for both pilots, unaffected bilateral control of shoulder and elbow movements and compromised control of wrist movements, while neither of the two maintained control over the fingers. Certified confirmation by their medical doctor of safety to participate in the Cybathlon event was requested and signed for both pilots, and insurance against accidents and injuries was taken, as per Cybathlon’s regulations. A safety and eligibility check was also conducted by the organizers the day before the competition.

Both participants maintain no control of the lower limbs and only limited control of the upper limbs. They are both able to stabilize their neck and head, but only P2 can also stabilize his trunk. Neither of our pilots carries pacemakers or other implants, suffers epilepsy or cyber-sickness, or needs respiratory assistance. They both use other advanced AT in their daily lives, like driving aids and speech-to-text software. P1 had several years prior participated in the MI BCI studies reported in [35,51,71], while P2 was BCI naive at the onset of his Cybathlon training. Informed consents have been signed in accordance with the Declaration of Helsinki, and their participation in the training sessions as well as in the competition has been approved by the Swiss committees for ethics in human research (protocol number PB_2017–00295, 20/15 CCVEM of the Cantonal Committee of Vaud, Switzerland for ethics in human research, CER-VD).

### Cybathlon BCI race

The Cybathlon competition comprised six different disciplines, each concerning a different type of AT (functional electrical stimulation, powered arm and leg prostheses, exoskeletons, wheelchairs, and BCI) [43]. The BCI race [8] consisted of (up to) four brain-controlled avatars, each actuated by a disabled pilot by means of mental commands, so as to reach the finishing line of a virtual race game called “Brain Runners” ahead of its opponents. Avatars would by default proceed at slow speed towards the finish line. The BCI pilot should be able to forward three mental commands to his/her avatar (spin, jump over prickles, slide under electrical rays), each of which would accelerate it only when issued while the avatar was traversing the corresponding color-coded track segment called “pad” (spin on cyan, jump on magenta, and slide on yellow pads). The acceleration effect would last until the avatar reached the beginning of the next pad or upon reception of a following erroneous command overriding the user’s correct command (whichever happened first). In addition to these three “action” pads, a fourth type (white pads) required “idling” to avoid any command delivery. A misplaced command, including false positives on the white pads, would slow down the pilot's progress towards the finish line of the track for 4 s (this timer would reset if another erroneous command or false positive was received in the meantime), until the beginning of the next pad or a following correct command overriding the erroneous one (whichever happened first). Besides the accelerating/slowing down behavior of the avatar, a thunder of the corresponding color briefly appearing over the avatar’s head would inform the pilot of the command currently sent. Support of at least one mental command was required to participate in the competition.

The standard track was composed of 16 pads (four of each type) randomly arranged so that the order of pads was not known to the competitors beforehand and was different for every race. The starting and finishing lines were situated on two additional white pads, so that the total distance to be covered by the pilots’ avatars was 500 virtual meters. The lower bound of race completion time on this track (i.e., the one achieved with an ideal input) is 54 s. The corresponding upper bound (continuous erroneous delivery) is 327 s, although only times below 240 s were considered valid in the actual competition. Since the avatars would proceed by default forward at a low “baseline” speed, the race completion time in case of no input whatsoever would be 162 s. The equivalent minimum, no-response, and maximum crossing times for the action pads were 2 s, 11 s, and 19 s, respectively. Hence, 11–19 s is the time frame within which a user is required to forward a correct command, with delivery speed being equally important to command accuracy. The minimum and maximum crossing times for the white pads were 5.5 s and 19 s, respectively. The corresponding times for the starting white pad were 5 s and 13 s, while for the ending white pad 3 s and 10 s.

The exclusion criteria for the technology provider dictated the use of noninvasive interfaces, while visual, tactile, or any kind of BCI feedback other than the one provided directly by the Brain Runners graphical user interface was prohibited, effectively excluding synchronous, stimuli-driven BCI paradigms like P300 and SSVEP. Besides the Brain Tweakers, another 10 international franchises participated in the tournament. From the 13 originally declared teams, two teams withdrew and one participated “out of competition” due to pilot ineligibility. The BCI race tournament format involved, initially, four qualification races (morning). The pilots marking the best four race completion times would qualify to Final A (afternoon) and compete for one of the 3 medals (gold, silver, and bronze), while the second-best four times would compete for places 5–8 in Final B. The event took place in a crowded, sold-out arena in front of a loud audience of roughly 4,600 spectators. A mock-up “rehearsal” event was held on July 14th, 2015, to ensure the best possible preparation for both the teams and the organizers.

### Training modalities, periods, and locations

Our mutual (subject and machine) learning approach involved three different training modalities aiming to establish, on the one hand, the end-users’ best possible control over spontaneous modulation of their SMRs by means of MI tasks and, on the other hand, their fast and accurate recognition on the part of the trained MI BCI algorithm. MI is defined as the mental rehearsal of a movement without overt motor output [72]. For MI tasks related to completely paralyzed limbs (legs for both P1 and P2), our pilots were instructed to attempt the corresponding movement, otherwise imagination suppressing any overt motor act was requested. During the competition, judges controlled for violations of the latter prerequisite.

Initially, open-loop, “offline” training (MI without real-time feedback) was applied, in order to exploit and calibrate the BCI on spontaneous SMR modulations the users could already elicit for the tested MI tasks. In this phase, we have mainly explored the existence of distinct brain patterns corresponding to right hand, left hand, both hands, both feet MI, and rest. Subsequently, offline runs were limited to both hands and both feet MI, which both our pilots were found to optimally modulate, so as to collect “clean” data for updating the BCI algorithm’s parameters. P1 has also unsuccessfully tried imagination of tongue movement, as well as “word” and “mathematical association” mental tasks.

Closed-loop, “online” sessions followed, where the pilots proceeded with real-time BCI control of a conventional, continuous visual feedback cursor targeting the enhancement of the patterns' discriminancy in an operant conditioning fashion (feedback training), while the BCI parameters were later recalibrated to better reflect the evolving brain patterns with the derived EEG data. Online runs were mainly conducted using the discriminant (coincidentally, for both our pilots) both hands and both feet MI tasks (2-class). P1 attempted to operate a 3-class online modality (left hand, right hand, feet MI) for a few sessions. More details on the visual interface of these two modalities and exactly how the BCI feedback cursor is driven by the BCI algorithm, can be found in section “BCI implementation” in Appendix A of [51], as well as in S1 Movie.

The third and latest stage was dominated by actual racing with the training version of the Brain Runners game delivered to the contestants so that our pilots could get accustomed to the real application's demands, in which one had to rely solely on the discrete feedback embedded into the cluttered graphics of the game itself. Offline, online, and racing runs were often interleaved (S2 Table) in order to make these transitions smoother. For the first racing runs only, we allowed our pilots to also observe the visual BCI feedback. During race training, our pilots would generally compete against the “bot” avatar option provided by the game. The “skill level” of this bot competitor was gradually increased to increasingly challenge our pilots. The racing track was randomized for each race, simulating the actual Cybathlon conditions.

Prior to and including the competition day, P1 received 35 training sessions within the period April–October 2016, while P2 underwent 16 sessions from July–October 2016, both in an individualized and flexible schedule (approximately twice a week), which was intensified as the competition day was approaching. P1 executed in total 40 offline, 12 online, and 182 race runs, while P2 did 15, 19, and 57 runs, respectively (S2 Table). All training sessions took place at the pilots’ homes under the supervision of one or two BCI engineers, except for two distinct sessions accommodated in the laboratory, where our two pilots competed against each other in the presence of a crowd of spectators, so as to simulate and get used to the special conditions they would cope with on the competition day.

### BCI implementation

The Brain Tweakers participation in the Cybathlon BCI race relied on the EEG-based MI BCI design previously developed in CNBI, which had already been shown to allow end-users to successfully operate a number of BCI prototypes [51]. For both user training and competitive racing, EEG was acquired with a lightweight, 16-channel g.USBamp amplifier (g.Tec medical engineering, Schiedelberg, Austria). The experimental setup during training additionally consisted of one laptop running the BCI algorithms and another one running the Brain Runners game. In the actual competition, the latter was substituted by the competition’s dedicated monitor displaying the race from each pilot’s individual viewpoint.

The EEG signal was recorded at 512 Hz sampling rate, band-pass filtered within 0.1 and 100 Hz, and notch-filtered at 50 Hz. The monitored EEG channels were selected so as to adequately cover the sensorimotor cortex (S3A Fig). The signal was spatially filtered with a Laplacian derivation, and the power spectral density (Welch periodogram) of each channel was computed with 2 Hz resolution in 1 s-long windows sliding every 62.5 ms. Feature selection was performed by ranking the candidate spatiospectral features according to discriminant power, calculated through canonical variate analysis, eventually manually selecting the most discriminant and neurophysiologically relevant ones. A Gaussian classifier outputting a probability distribution over two MI tasks was used to classify the consecutive feature vectors in real time. The Gaussian classifier was trained with a gradient-descent supervised learning approach using the labeled MI datasets resulting from the aforementioned training protocols. The samples with “uncertain” probability distributions (where the maximum probability does not exceed a certain threshold) were rejected, while the remaining ones were fed to an evidence accumulation module smoothing the classifier output by means of a leaky integrator (exponential smoothing). A final decision is emitted by the BCI system once the pilot is able to push the integrated probabilities of some mental class to reach a configurable decision threshold by consistently performing the corresponding MI task, thus forwarding the associated command to his/her Brain Runners avatar. Upon delivery of a BCI command, the integrated probabilities are reset to the uniform distribution so as to start an unbiased new trial. A refractory period of 1 s was set in between consecutive commands. An artifact rejection scheme would block the BCI output once ocular and facial muscle artifacts were detected. A more detailed description of all the above methods is provided in Appendix A of [51] and the references therein.

### Artifact rejection scheme

Under the Cybathlon BCI race regulations, all teams should embed an artifact removal or rejection framework into their BCI system, ensuring that the pilot’s avatar is actuated by means of brain signals only, without interference from other signals originating from muscle activity or at the level of the peripheral nervous system (PNS). Thus, the Brain Tweakers artifact control scheme targeted the detection of electrooculogram (EOG) and facial electromyogram (EMG) signals, upon which the BCI output was blocked for a configurable interval preventing any outgoing command towards the pilot’s BrainRunners avatar. Respecting the need for a minimally obtrusive setup, only four electrode/sensor pairs are employed to extract two bipolar EOG channels, by means of a second synced g.USBamp device. One sensor is placed on either eye canthus, a third one on the pilot’s nasion bone, while the last sensor acts as the reference and is placed on the pilot’s forehead (S3B Fig). In sync with the EEG acquisition, EOG signals are acquired at 512 Hz in frames of 62.5 ms. Artifact detection is performed separately on each consecutive frame, resulting in very fluid and responsive detection of artifact onset and offset. For each frame, the original channels EOG_i_, i ∈ [1, 4] are combined to form a horizontal (EOG_h_ = EOG_1_ − EOG_3_) and a vertical (EOG_v_ = EOG_2_ − (EOG_1_ + EOG_3_)/2) channel, specializing in capturing horizontal and vertical eye movements, respectively. The average of all channels was also extracted and monitored, as it is particularly sensitive to eye blinks and intense facial muscle flexions. All channels were band-pass filtered between 1 and 10 Hz with a second-order Butterworth filter and rectified. Finally, the processed channel frames are compared against a common configurable threshold. The individual frame decision was 1 when any of the processed samples within the current frame exceeds the threshold and 0 otherwise. The final artifact detection module would communicate an artifact onset event to the game controller upon a frame decision transition from 0 to 1, signaling the blocking of the BCI output. An artifact offset event lifting the BCI command blocking was issued after a configurable timeout since the latest artifact onset detection.

Since a distinct feature of this study is BCI operation in real-world conditions, where arbitrary artifact contamination is common, we opted for transparency to always report imaging (in particular, discriminancy) results directly on raw data. S4 Fig illustrates that three lateral channels of P2 (of which the only selected channel is CP3) exhibit an unidentified high-frequency component late in training. This effect only concerns two of the features selected for P2’s BCI control (CP3/30 Hz and CP3/32 Hz) and approximately one-fourth of the executed runs. Applying the artifact removal algorithm FORCe [73] effectively eliminates this component (S1 Fig). Importantly, S5 Fig shows that the discriminancy of these two selected features constitutes a genuine EEG MI correlate, as it is present at different sessions of this pilot’s training in the absence of the potentially artifactual component. Of note, all effects shown in the manuscript regarding discriminancy (including correlations with the amount of training, race completion time, pad crossing time, and BCI accuracy) still hold for both pilots after FORCe artifact removal, as shown in S6 Fig for pilot P2.

### Game control paradigm

The game control paradigm defines the way the pilot’s motor imaginations translate into avatar actions through the emitted BCI commands. Several control paradigms have been designed and tested throughout the training period in close cooperation between the Brain Tweakers researchers and pilots. Initially, we explored the straightforward option of a 3-class BCI (paradigm 1) employing right hand, left hand, and both feet MI. Thereby, each BCI command was directly mapped to a certain avatar action (right hand MI to spin, both feet MI to jump, and left hand MI to slide). A 2-class BCI (paradigm 2) preserving the previous mapping but leaving the slide command unsupported was also tested. Given the unsatisfactory outcome of these two approaches, another two paradigms were designed, both investigating well-known human–computer interaction principles for supporting all three avatar commands given only a binary input. Specifically, the two separable MI tasks (both hands and feet MI for both our pilots) were again directly mapped to the spin and jump avatar actions. Additionally, paradigm 3 would make the avatar slide after a configurable period of INC. Paradigm 4, on the other hand, would trigger sliding when two consecutive commands of different types (i.e., a spin/jump or jump/spin pair) were forwarded within a configurable interval. Paradigm 4 was adopted for the competition.

In all four tested control paradigms, “idling” is achieved through the “resting” mental task, where the pilot is deliberately not engaging in any MI task (INC). Since the BCI classifier is continuously (every 62.5 ms, i.e., at 16 Hz) outputting a probability distribution over the MI mental classes (not including the resting state), INC is achieved through a statistical approach in which, thanks to the evidence accumulation module and the BCI’s optimized parametrization (decision and sample rejection thresholds, smoothing parameter), a BCI command is only forwarded when the user is consistently performing the associated MI. Otherwise, the integrated probabilities will tend to fluctuate below the decision thresholds, avoiding any command forwarding [51].

### Evaluation metrics and data

The race completion and the pad crossing times are measured in seconds (s). BCI performance is quantified through BCI command accuracy, which is the percentage of pads in a race in which the correct command has been delivered within the given time frame (i.e., while the pilot’s avatar is on the particular pad). Pad crossing times (“time on pad” metric) are reported to simultaneously evaluate BCI command accuracy and delivery speed. The total BCI command accuracy in a race is computed as the average per command accuracies (class-specific true positive rates). For the white pads, an equivalent accuracy metric (true negative rate) is calculated as the percentage of white pads in the race that the pilot managed to cross without delivering any command. Finally, discriminancy of a given spatiospectral EEG feature (corresponding to a certain EEG channel and a frequency band) for two mental classes is quantified through Fisher score as $FS=\frac{\left|\mu_{1}-\mu_{2}\right|}{\sqrt{{s_{1}}^{2}+{s_{2}}^{2}}}$, where *μ*_1_,*μ*_2_ are the means and *s*_1_,*s*_2_ are the standard deviations of this feature’s sample values for mental class 1 (Both Hands) and 2 (Both Feet), respectively. Discriminancy over two large and physiologically relevant to MI topographic (lateral: FC3, FC4, C3, C4, CP3, CP4 and medial: FCz, Cz, CPz) and spectral (μ: 8–14 Hz and β: 22-32Hz) regions is computed as the average Fisher score of all features corresponding to the channels and frequency bands of the regions in question. S2 Table presents the list of sessions executed and the type of data acquired in each.

### Statistical analysis

Point estimates are reported using averages or medians and dispersions as standard deviation or 25th and 75th percentiles, when the underlying distribution is normal or skewed, respectively. Training effects are shown by reporting Pearson correlation coefficients and their significance at the 95% confidence interval through Student *t* test distribution. The same type of correlation is employed to study the relationship of the application, machine, and subject learning evaluation metrics. Additionally, the first and last four sessions are compared and tested for significant differences at the 95% confidence interval using unpaired, two-sided Wilcoxon nonparametric rank-sum tests.

### Questionnaire on user training

A short questionnaire requesting information on essential details of the user and system training methodology adopted has been addressed to all competing teams in an attempt to identify critical elements of successful training strategies. The questionnaire consisted of the following 5 questions:

1. What was the total duration of your pilot's training (in weeks or months)?
2. What was the intensity of your user training approach (sessions per week, on average)?
3. What was the total number of training sessions until the Cybathlon BCI race event?
4. How often was the BCI decoder/classifier retrained?
5. Did BCI recalibration involve only classifier parameter update or also feature re-selection?

Teams BrainGain [74], Athena-Minerva [75,76], NeuroCONCISE [77,78], OpenBMI, Mahidol BCI, and MIRAGE91 [37] have provided the requested info.

## Supporting information

S1 Fig BCI feature discriminancy maps per run (N) averaged for each training month. Bright color indicates high discriminancy between Both Hands and Both Feet MI tasks employed by both pilots (P1 top, P2 bottom). The discriminancy of each feature (channel-frequency pair) is quantified as the Fisher score of the EEG signal's power spectral density distributions for these two mental classes. Raw data have been cleaned with the artifact removal algorithm FORCe [73]. S1 Fig data is located at https://doi.org/10.5281/zenodo.1205852. BCI, brain–computer interface; EEG, electroencephalography; MI, motor imagery.
(TIF)

S2 Fig BCI feature discriminancy per training modality. Topographic maps of discriminancy per training modality on the 16 EEG channel locations over the sensorimotor cortex monitored. Bright color indicates high discriminancy between Both Hands and Both Feet MI tasks employed by both pilots (P1 top, P2 bottom). The discriminancy of each channel is quantified as the Fisher score of the EEG signal's power spectral density distributions for these two mental classes in the high β band (22–32 Hz) on this channel. Each map illustrates local Fisher scores (with interchannel interpolation) averaged over all runs of the supertitled modality. S2 Fig data is located at https://doi.org/10.5281/zenodo.1205860. BCI, brain–computer interface; EEG, electroencephalography; MI, motor imagery.
(TIF)

S3 Fig Electrode configurations. **(A)** EEG channel configuration over 16 locations of the sensorimotor cortex according to the international 10–20 system. **(B)** EOG electrode configuration on the pilot’s right and left canthi, nasion, and forehead for the detection of ocular and facial muscle artifacts. EEG, electroencephalography; EOG, electrooculogram.
(TIF)

S4 Fig BCI feature discriminancy maps per run (N) averaged for each training month. Bright color indicates high discriminancy between Both Hands and Both Feet motor imagery tasks employed by both pilots (P1 top, P2 bottom). The discriminancy of each feature (channel–frequency pair) is quantified as the Fisher score of the EEG signal's power spectral density distributions for these two mental classes. Discriminancy is computed on raw data without artifact removal. S4 Fig data is located at https://doi.org/10.5281/zenodo.1213033. BCI, brain–computer interface; EEG, electroencephalography.
(PNG)

S5 Fig BCI feature discriminancy maps for three typical BCI sessions of pilot P2 in August, September, and October after artifact removal with FORCe. Bright color indicates high discriminancy between Both Hands and Both Feet motor imagery tasks employed by pilot P2. The discriminancy of each feature (channel–frequency pair) is quantified as the Fisher score of the EEG signal's power spectral density distributions for these two mental classes. These three maps show that features CP3/30 Hz and CP3/32 Hz selected for control correspond to real EEG MI correlates, as they remain discriminant in the absence of the potentially artifactual high-frequency component. S5 Fig data is located at https://doi.org/10.5281/zenodo.1213164. BCI, brain–computer interface; EEG, electroencephalography; MI, motor imagery.
(PNG)

S6 Fig BCI feature discriminancy for pilot P2 after artifact removal with FORCe. **(A)** Topographic maps of discriminancy per training month on the 16 EEG channel locations over the sensorimotor cortex monitored. Bright color indicates high discriminancy between Both Hands and Both Feet MI tasks employed by pilot P2. The discriminancy of each channel is quantified as the Fisher score of the EEG signal's power spectral density distributions for these two mental classes in the high β-band (22–32 Hz) within each run. Each map illustrates local Fisher scores (with interchannel interpolation) averaged over all runs within the supertitled month. **(B)** Average medial (blue, channels: FCz, Cz, CPz) and lateral (red, channels: FC3, C3, CP3, FC4, C4, CP4) discriminancy for all performed offline, online, and racing runs of pilot P2. The corresponding linear fits and Pearson correlation coefficients (significance tested with Student *t* test distribution) are reported to indicate training effects. Vertical dashed lines indicate the training session where each run took place. **(C)** Average and standard deviations of medial region (blue) and lateral region (red) discriminancy within the first and last four runs of training for pilot P2. Statistically significant differences are shown with two-sided Wilcoxon ranksum tests, (***): *p* < .001. S6 Fig data is located at https://doi.org/10.5281/zenodo.1213100, https://doi.org/10.5281/zenodo.1213106, https://doi.org/10.5281/zenodo.1213108. BCI, brain–computer interface; EEG, electroencephalography; MI, motor imagery.
(PNG)

S1 Table User-training methodology details of the Cybathlon BCI race competitors. BCI, brain–computer interface.
(DOCX)

S2 Table Training session information. The table presents the date of all executed training sessions for both pilots and the number and type of runs performed in each session and reported here. Asterisks indicate one or more runs have been lost due to technical failure or bad maintenance.
(DOCX)

S1 Movie Typical race training session of pilot P1. (MP4)

## Abbreviations

AI: artificial intelligence
ALS: amyotrophic lateral sclerosis
ASIA: American Spinal Injury Association
AT: assistive technology
BCI: brain–computer interface
EEG: electroencephalography
EMG: electromyogram
EOG: electrooculogram
ERD/ERS: event-related desynchronization/synchronization
IC: intentional control
INC: intentional non-control
MI: motor imagery
PNS: peripheral nervous system
SCI: spinal cord injury
SMR: sensorimotor rhythm
SNR: signal-to-noise ratio
SSVEP: steady-state visually evoked potentials
ZHdK: Zurich University of the Arts
