# Supplementary figures and images for: The Cybathlon BCI race: Successful longitudinal mutual learning with two tetraplegic users
Source: PLoS Biol. 2018 May 10;16(5):e2003787. doi: 10.1371/journal.pbio.2003787 (PMC5944920; doi:10.1371/journal.pbio.2003787)

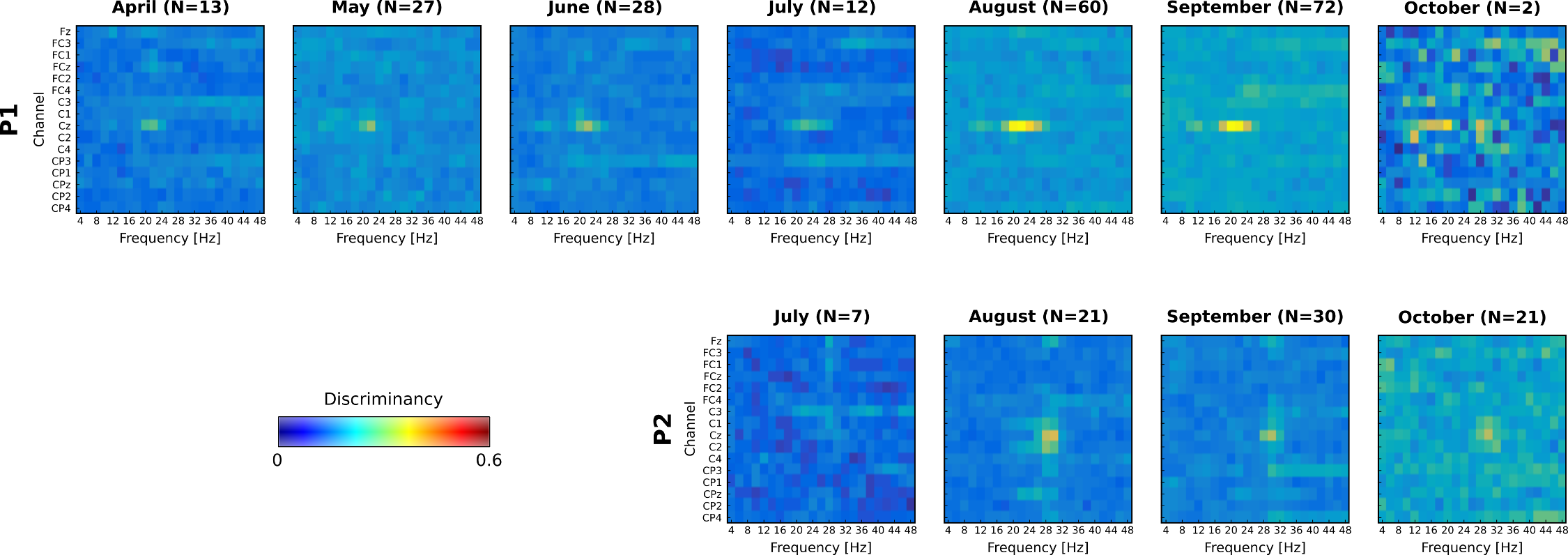

Supplement: S1 Fig — Bright color indicates high discriminancy between Both Hands and Both Feet MI tasks employed by both pilots (P1 top, P2 bottom). The discriminancy of each feature (channel-frequency pair) is quantified as the Fisher score of the EEG signal's power spectral density distributions for these two mental classes. Raw data have been cleaned with the artifact removal algorithm FORCe [73]. S1 Fig data is located at https://doi.org/10.5281/zenodo.1205852. BCI, brain–computer interface; EEG, electroencephalography; MI, motor imagery. (TIF) [file pbio.2003787.s001.tif]

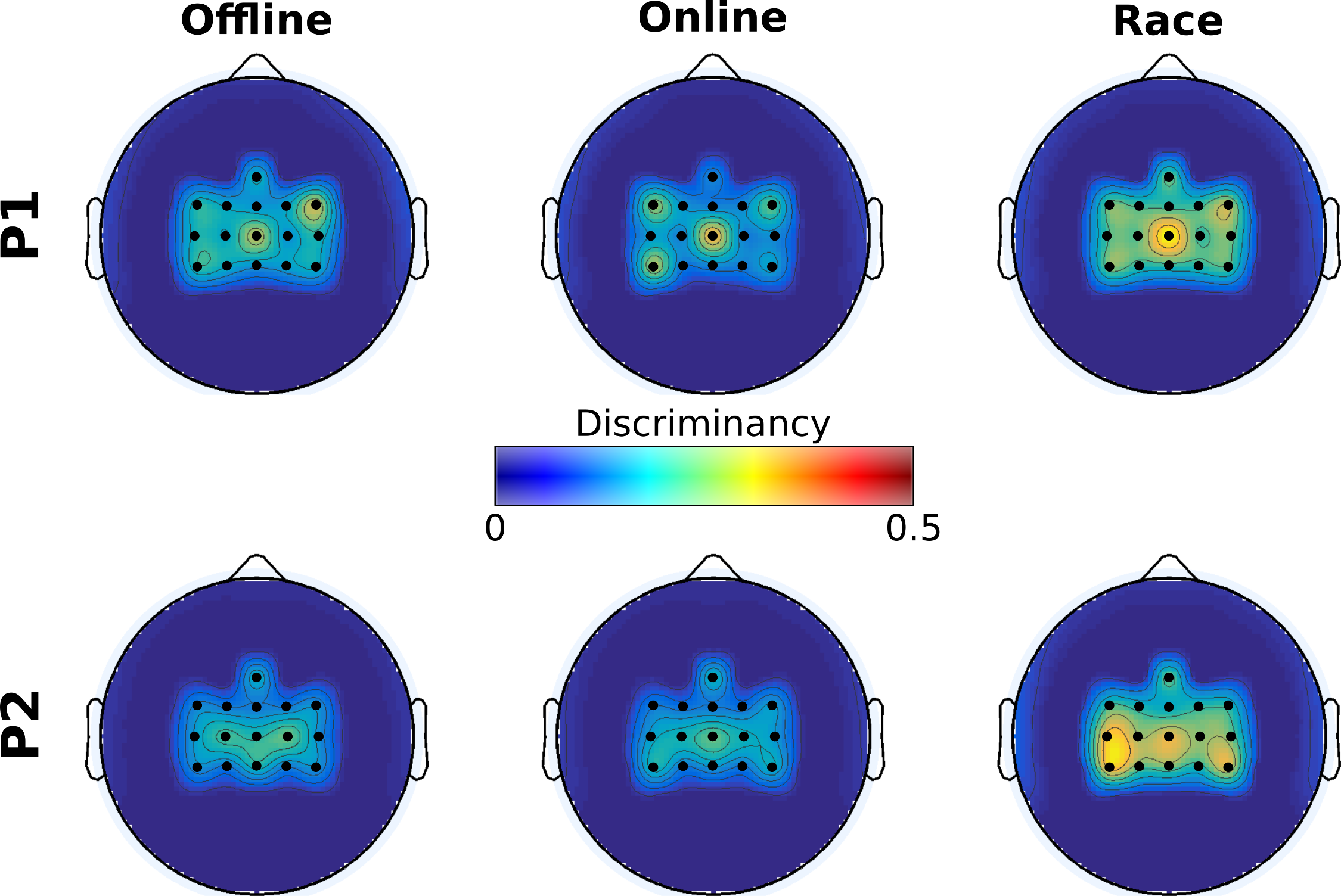

Supplement: S2 Fig — Topographic maps of discriminancy per training modality on the 16 EEG channel locations over the sensorimotor cortex monitored. Bright color indicates high discriminancy between Both Hands and Both Feet MI tasks employed by both pilots (P1 top, P2 bottom). The discriminancy of each channel is quantified as the Fisher score of the EEG signal's power spectral density distributions for these two mental classes in the high β band (22–32 Hz) on this channel. Each map illustrates local Fisher scores (with interchannel interpolation) averaged over all runs of the supertitled modality. S2 Fig data is located at https://doi.org/10.5281/zenodo.1205860. BCI, brain–computer interface; EEG, electroencephalography; MI, motor imagery. (TIF) [file pbio.2003787.s002.tif]

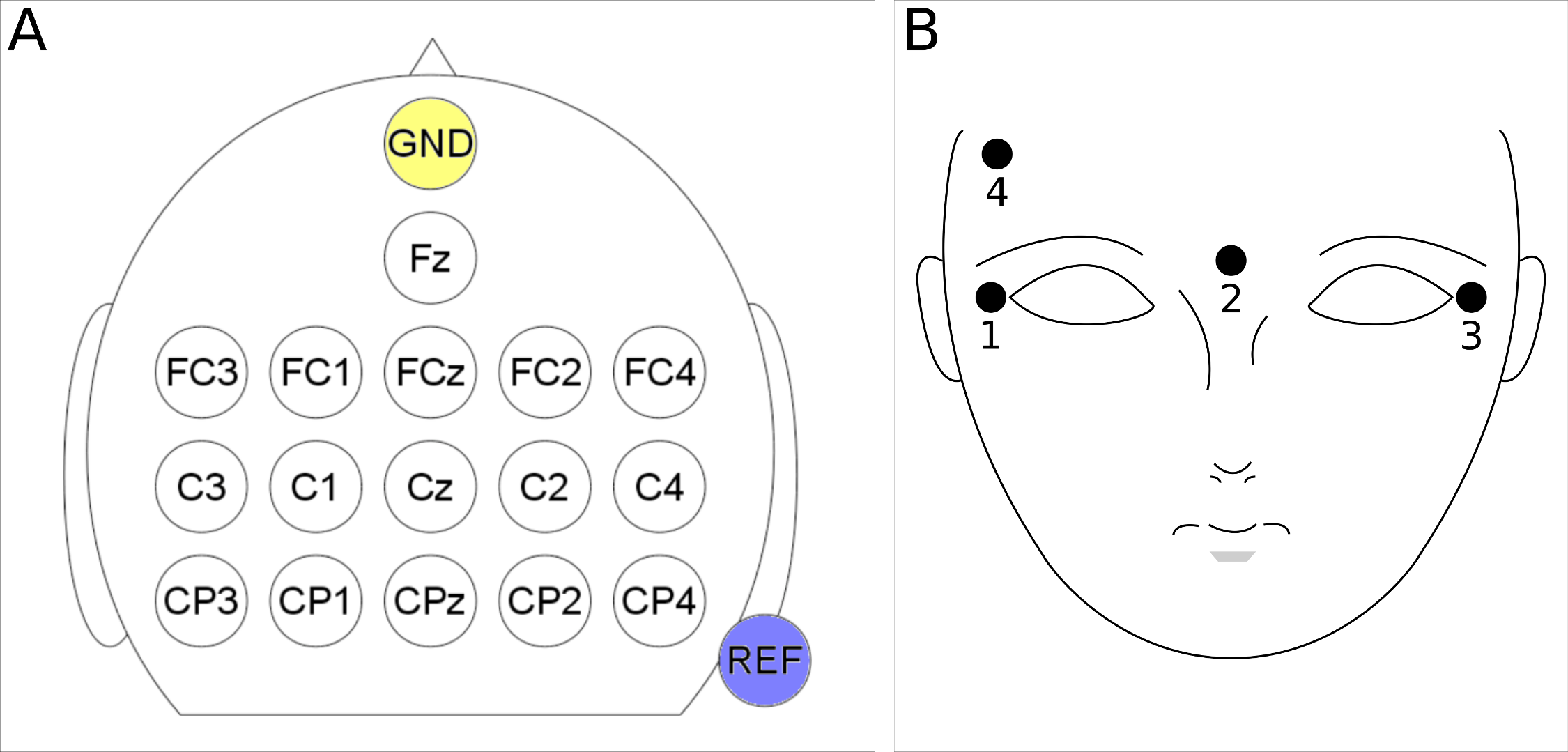

Supplement: S3 Fig — (A) EEG channel configuration over 16 locations of the sensorimotor cortex according to the international 10–20 system. (B) EOG electrode configuration on the pilot’s right and left canthi, nasion, and forehead for the detection of ocular and facial muscle artifacts. EEG, electroencephalography; EOG, electrooculogram. (TIF) [file pbio.2003787.s003.tif]

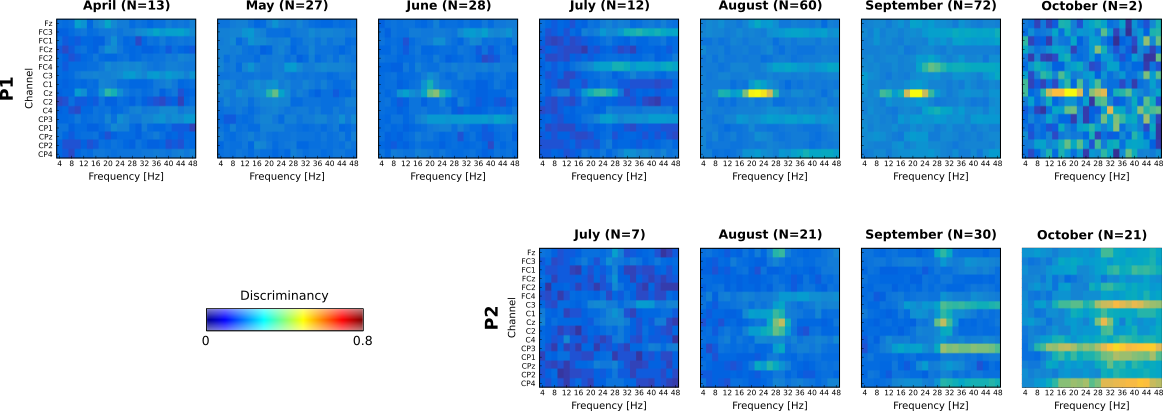

Supplement: S4 Fig — Bright color indicates high discriminancy between Both Hands and Both Feet motor imagery tasks employed by both pilots (P1 top, P2 bottom). The discriminancy of each feature (channel–frequency pair) is quantified as the Fisher score of the EEG signal's power spectral density distributions for these two mental classes. Discriminancy is computed on raw data without artifact removal. S4 Fig data is located at https://doi.org/10.5281/zenodo.1213033. BCI, brain–computer interface; EEG, electroencephalography. (PNG) [file pbio.2003787.s004.png]

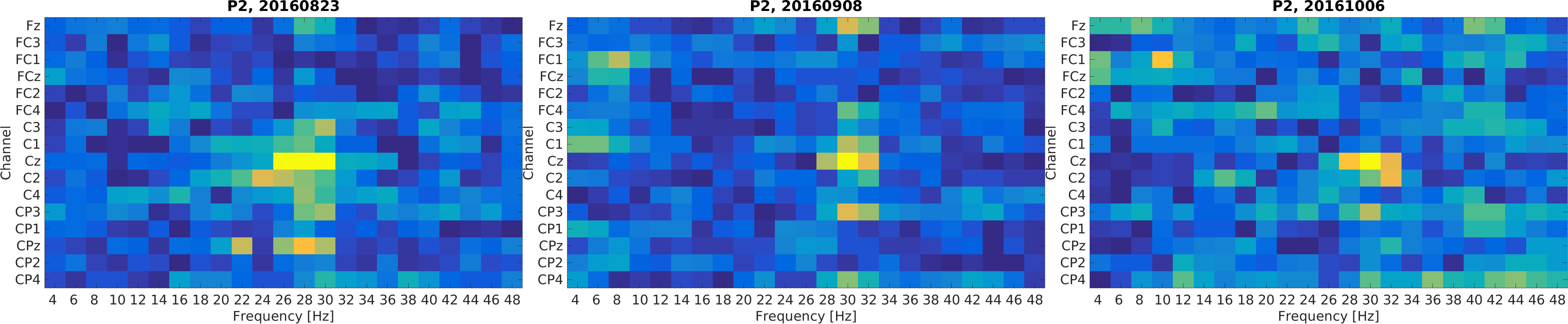

Supplement: S5 Fig — Bright color indicates high discriminancy between Both Hands and Both Feet motor imagery tasks employed by pilot P2. The discriminancy of each feature (channel–frequency pair) is quantified as the Fisher score of the EEG signal's power spectral density distributions for these two mental classes. These three maps show that features CP3/30 Hz and CP3/32 Hz selected for control correspond to real EEG MI correlates, as they remain discriminant in the absence of the potentially artifactual high-frequency component. S5 Fig data is located at https://doi.org/10.5281/zenodo.1213164. BCI, brain–computer interface; EEG, electroencephalography; MI, motor imagery. (PNG) [file pbio.2003787.s005.png]

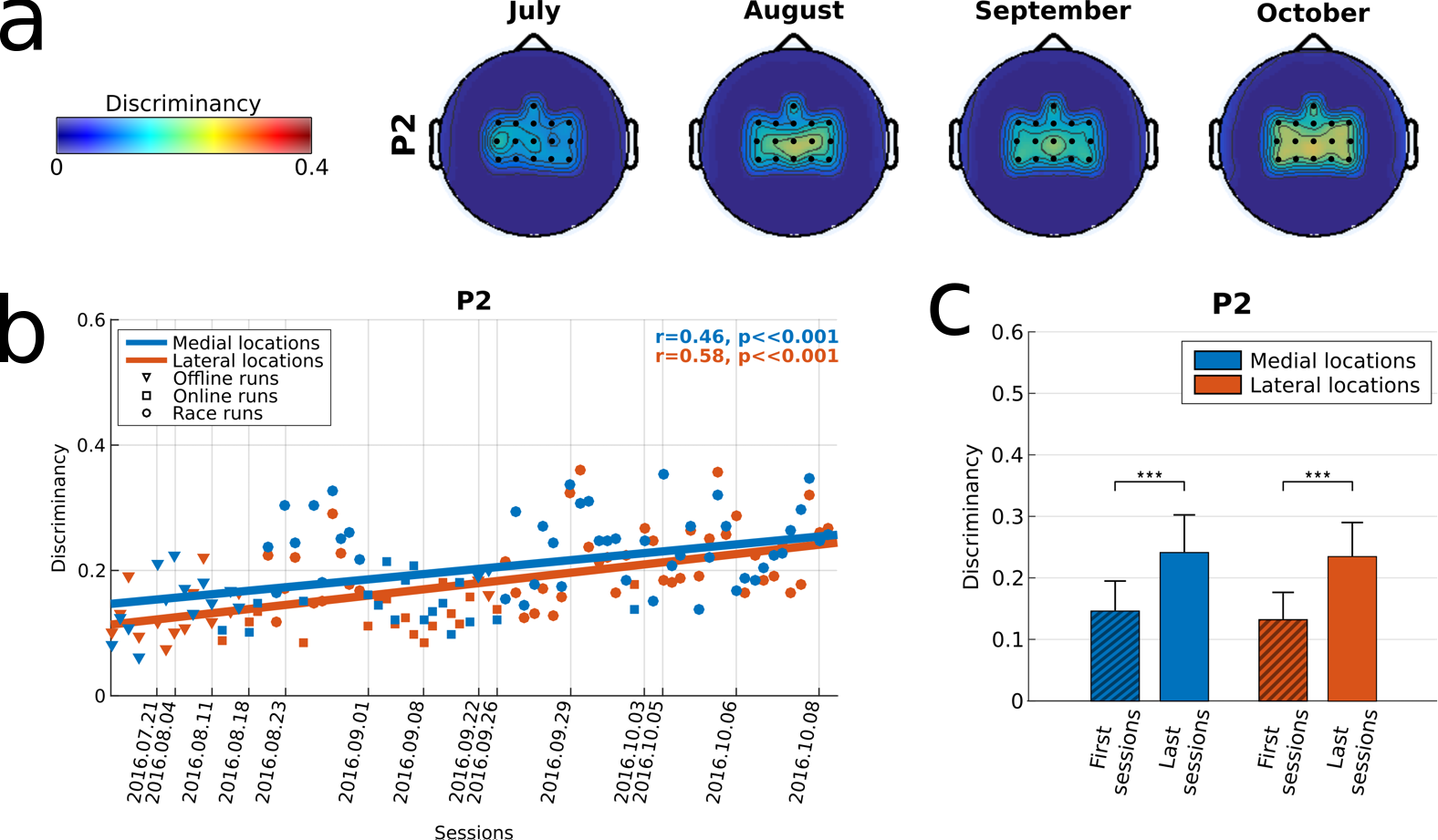

Supplement: S6 Fig — (A) Topographic maps of discriminancy per training month on the 16 EEG channel locations over the sensorimotor cortex monitored. Bright color indicates high discriminancy between Both Hands and Both Feet MI tasks employed by pilot P2. The discriminancy of each channel is quantified as the Fisher score of the EEG signal's power spectral density distributions for these two mental classes in the high β-band (22–32 Hz) within each run. Each map illustrates local Fisher scores (with interchannel interpolation) averaged over all runs within the supertitled month. (B) Average medial (blue, channels: FCz, Cz, CPz) and lateral (red, channels: FC3, C3, CP3, FC4, C4, CP4) discriminancy for all performed offline, online, and racing runs of pilot P2. The corresponding linear fits and Pearson correlation coefficients (significance tested with Student t test distribution) are reported to indicate training effects. Vertical dashed lines indicate the training session where each run took place. (C) Average and standard deviations of medial region (blue) and lateral region (red) discriminancy within the first and last four runs of training for pilot P2. Statistically significant differences are shown with two-sided Wilcoxon ranksum tests, (***): p < .001. S6 Fig data is located at https://doi.org/10.5281/zenodo.1213100, https://doi.org/10.5281/zenodo.1213106, https://doi.org/10.5281/zenodo.1213108. BCI, brain–computer interface; EEG, electroencephalography; MI, motor imagery. (PNG) [file pbio.2003787.s006.png]
