# Supplementary material for: The Cybathlon BCI race: Successful longitudinal mutual learning with two tetraplegic users
Source: PLoS Biol. 2018 May 10;16(5):e2003787. doi: 10.1371/journal.pbio.2003787 (PMC5944920; doi:10.1371/journal.pbio.2003787)
Supplement: S1 Table — BCI, brain–computer interface. (DOCX) [file pbio.2003787.s007.docx]

**S1 Table.** User-training methodology details of the Cybathlon BCI race competitors.

| **Team** | **Duration** | **# Sessions** | **Intensity** | **Recalibration** | **Feature re-selection** |
| --- | --- | --- | --- | --- | --- |
| BrainGain [74] | 1.5 months | 14 | 2 / week | Every session  and right before official races | No |
| Athena-Minerva [75,76] | 18 months | N/A | 1 / 2 weeks | Every session | Yes |
| OpenBMI | 3 months | 14 | 1-2 / week | Every session | Only first 6 weeks |
| NeuroCONCISE [77,78] | 1 month | 9 (+10 in 2011) | 2-3 / week | Every session | Yes |
| Mahidol BCI | 3 months | 26 | 2 / week  4 in last week | At least once per week | Yes |
| MIRAGE91 [37] | ~12 months | ~35 | 1 / month  2 / day in last week | Every session | No |
| Brain Tweakers | ~7 months (P1) ~4 months (P2) | 35 (P1)  16 (P2) | ~2 / week | Once | Once |
