# Supplementary material for: The Cybathlon BCI race: Successful longitudinal mutual learning with two tetraplegic users
Source: PLoS Biol. 2018 May 10;16(5):e2003787. doi: 10.1371/journal.pbio.2003787 (PMC5944920; doi:10.1371/journal.pbio.2003787)
Supplement: S2 Table — The table presents the date of all executed training sessions for both pilots and the number and type of runs performed in each session and reported here. Asterisks indicate one or more runs have been lost due to technical failure or bad maintenance. (DOCX) [file pbio.2003787.s008.docx]

**S2 Table.** Training session information. The table presents the date of all executed training sessions for both pilots and the number and type of runs performed in each session and reported here. Asterisks indicate one or more runs have been lost due to technical failure or bad maintenance.

| **P1** | | | | | **P2** | | | | |
| --- | --- | --- | --- | --- | --- | --- | --- | --- | --- |
| **Session**  **#** | **Session**  **Date** | **Offline** | **Online** | **Race** | **Session**  **#** | **Session**  **Date** | **Offline** | **Online** | **Race** |
| 1 | 04/04 | 5 | 1 | 0 | 1 | 14/07 | 4 | 1* | 0 |
| 2 | 08/04 | 3 | 0 | 0 | 2 | 21/07 | 2 | 0 | 0 |
| 3 | 18/04 | 2 | 0 | 0* | 3 | 04/08 | 4 | 0 | 0 |
| 4 | 29/04 | 0 | 0 | 2* | 4 | 11/08 | 3 | 1* | 0 |
| 5 | 02/05 | 0 | 0 | 5 | 5 | 18/08 | 0 | 2* | 2 |
| 6 | 09/05 | 0 | 0 | 8 | 6 | 23/08 | 0 | 1 | 8 |
| 7 | 20/05 | 1 | 0 | 3 | 7 | 25/08 | 0 | 0 | 7* |
| 8 | 27/05 | 1 | 0 | 9 | 8 | 01/09 | 0 | 6 | 0 |
| 9 | 03/06 | 0 | 0 | 9 | 9 | 08/09 | 0 | 6 | 0 |
| 10 | 17/06 | 2 | 2 | 3 | 10 | 22/09 | 2 | 0 | 5* |
| 11 | 24/06 | 5 | 0 | 0 | 11 | 26/09 | 0 | 1* | 7 |
| 12 | 30/06 | 6 | 1 | 0 | 12 | 29/09 | 0 | 1 | 7 |
| 13 | 04/07 | 2 | 5 | 0 | 13 | 03/10 | 0 | 0 | 2 |
| 14 | 07/07 | 1* | 1 | 0* | 14 | 05/10 | 0 | 0 | 8 |
| 15 | 13/07 | 1 | 0 | 0* | 15 | 06/10 | 0 | 0* | 9 |
| 16 | 20/07 | 1 | 0 | 0* | 16 | 08/10 | 0 | 0 | 2 |
| 17 | 25/07 | 0* | 0* | 0 | **Total** | 16 | 15 | 19 | 57 |
| 18 | 26/07 | 0* | 0* | 0 |  |  |  |  |  |
| 19 | 27/07 | 1 | 0 | 0* |  |  |  |  |  |
| 20 | 03/08 | 1 | 0 | 0* |  |  |  |  |  |
| 21 | 10/08 | 1 | 0 | 11 |  |  |  |  |  |
| 22 | 15/08 | 0 | 0 | 10 |  |  |  |  |  |
| 23 | 17/08 | 3 | 0 | 7 |  |  |  |  |  |
| 24 | 24/08 | 0 | 0 | 11 |  |  |  |  |  |
| 25 | 25/08 | 0 | 0 | 16* |  |  |  |  |  |
| 26 | 31/08 | 0 | 0 | 16 |  |  |  |  |  |
| 27 | 07/09 | 0 | 0 | 10 |  |  |  |  |  |
| 28 | 09/09 | 4 | 0 | 7 |  |  |  |  |  |
| 29 | 14/09 | 0 | 0 | 0 |  |  |  |  |  |
| 30 | 21/09 | 0 | 0 | 10 |  |  |  |  |  |
| 31 | 22/09 | 0 | 0 | 16 |  |  |  |  |  |
| 32 | 28/09 | 0 | 1 | 9 |  |  |  |  |  |
| 33 | 30/09 | 0 | 1 | 14 |  |  |  |  |  |
| 34 | 06/10 | 0 | 0 | 9* |  |  |  |  |  |
| 35 | 08/10 | 0 | 0 | 2 |  |  |  |  |  |
| **Total** | 35 | 40 | 12 | 182 |  |  |  |  |  |
